# A method for synchronized use of EEG and eye tracking in fully immersive VR

**DOI:** 10.3389/fnhum.2024.1347974

**Published:** 2024-02-26

**Authors:** Olav F. P. Larsen, William G. Tresselt, Emanuel A. Lorenz, Tomas Holt, Grethe Sandstrak, Tor I. Hansen, Xiaomeng Su, Alexander Holt

**Affiliations:** ^1^Motion Capture and Visualization Laboratory, Department of Computer Science, Faculty of Information Technology and Electrical Engineering, Norwegian University of Science and Technology, Trondheim, Norway; ^2^Department of Neuromedicine and Movement Science, Faculty of Medicine and Health Sciences, Norwegian University of Science and Technology, Trondheim, Norway; ^3^Department of Acquired Brain Injury, St. Olav's University Hospital, Trondheim, Norway

**Keywords:** electroencephalography, eye-tracking, virtual reality, brain-computer interface, speller, synchronization, SSVEP

## Abstract

This study explores the synchronization of multimodal physiological data streams, in particular, the integration of electroencephalography (EEG) with a virtual reality (VR) headset featuring eye-tracking capabilities. A potential use case for the synchronized data streams is demonstrated by implementing a hybrid steady-state visually evoked potential (SSVEP) based brain-computer interface (BCI) speller within a fully immersive VR environment. The hardware latency analysis reveals an average offset of 36 ms between EEG and eye-tracking data streams and a mean jitter of 5.76 ms. The study further presents a proof of concept brain-computer interface (BCI) speller in VR, showcasing its potential for real-world applications. The findings highlight the feasibility of combining commercial EEG and VR technologies for neuroscientific research and open new avenues for studying brain activity in ecologically valid VR environments. Future research could focus on refining the synchronization methods and exploring applications in various contexts, such as learning and social interactions.

## 1 Introduction

In cognitive neuroscience research, the acquisition of multimodal physiological signals has gained prominence in exploring the relationships between behavior and associated cortical activity. This development is promoted by advances in computer science, increasing the accessibility and precision of associated technologies. Fields such as vision research, mobile brain imaging (MoBi), neurorehabilitation, and neuromarketing use multimodal physiological data, achieving ecologically valid insight into the underlying cortical processes (McMullen et al., 2014; King and Parada, 2021; Pereira et al., 2021; Zhu and Lv, 2023).

One frequently employed modality used within cognitive and neuroscience research is optical eye-tracking. This technique allows for the non-invasive tracking of eye movements and gaze fixations, as well as pupillometry using optical sensors like video cameras (Punde et al., 2017). Thus, eye-tracking is a valuable instrument for analyzing behavioral metrics and cognitive processes such as attention, cognitive workload, emotional processing, and memory (Lim et al., 2020; Ryan and Shen, 2020; Vehlen et al., 2021; Pradhan and Kumar, 2022). Beyond its research applications, optical eye-tracking finds utility in clinical contexts, such as systematically evaluating stroke-related neurologic deficits, aiding in designing treatment strategies and predicting therapy results (Kaiser et al., 2022). Besides eye-tracking specialized for medical and research applications, which achieve high temporal and spatial accuracy, the market has seen a proliferation of cost-effective commercial products (Kapp et al., 2021). In particular, current XR headsets often provide integrated eye-tracking, initially to enhance immersion in gaming experiences (Adhanom et al., 2023). However, open-source solutions exist to access the integrated eye-tracking system for other purposes directly (Tobii, 2023; ValveSoftware, 2023).

In addition to their eye-tracking capabilities, commercial Extended-Reality (XR) headsets are considered valuable tools to provide realistic and, thus, ecologically valid environments within the confines of a laboratory. The headset can generate the illusion of a three-dimensional world by providing distinct two-dimensional images for each eye. Additionally, the system actively tracks the user's head position and orientation in space, thus enhancing the immersive experience (Parsons, 2015). Hand-held controllers or hand-tracking technologies ultimately allow for interaction with the virtual environment (Buckingham, 2021). Such immersive displaying techniques increase the user's engagement toward the visual paradigm and allow for investigating cortical processes within ecologically valid environments (Parsons, 2015; Gall et al., 2021). Thus, it sees increased use for medical applications, such as motor rehabilitation and mental disorders (Srivastava et al., 2014; Feitosa et al., 2022).

Another commonly used modality for evaluating cognitive processes is electroencephalography (EEG). Recently, it has been used more frequently in combination with XR headset application, chosen for its mobility, real-time capabilities, and low-cost point (Ocklenburg and Peterburs, 2023). By amplifying the fast changes in electrical cortical activity recorded over electrodes attached non-invasively to the scalp, it becomes possible to derive corresponding cortical processes at high temporal resolution (Jackson and Bolger, 2014). Cognitive neuroscience research using EEG often utilizes specific stimuli or behavioral events to evoke an event-specific cortical activity known as event-related potential (ERP) (Luck, 2012). Given that these events occur within milliseconds, the setup demands exceptionally high temporal resolution. Furthermore, the ERPs mentioned were also used in medical applications, such as brain-computer interfaces (BCI). BCIs are often used as an assistive communication tool e.g., for patients with locked-in syndrome, such as in P300 spellers or steady-state visual evoked potential (SSVEP) spellers that allow patients to communicate based on their cortical response to a presented stimulus (Kundu and Ari, 2022). Eye tracking has been combined with EEG in several studies in neuroscience (Langer et al., 2017; Zhu and Lv, 2023), which often employed research-grade eye-tracking devices that enabled seamless integration into the EEG recording with high temporal and spatial accuracy. However, the question remains whether this can be done with easily accessible commercial eye-tracking devices, such as those integrated into XR headsets. The use of XR additionally allows for the generation of ecologically valid experimental tasks, which increases the applicability and generalizability of the results (Parsons, 2015).

To fully harness the capabilities of these combined technologies, a deep and comprehensive understanding of the technical challenges in synchronizing and integrating measurements is crucial. Although hybrid XR headsets with integrated eye-tracking and EEG are being developed, as Neurospec's DSI-VR300 and OpenBCI's Galea (AG, 2023; OpenBCI, 2023), their cost and lack of flexibility, such as lack of support to not manufacturer-endorsed applications or high-density EEG, render them at times ill-suited for a variety of research applications. Thus, various open-source methods were developed recently to allow for synchronized multimodal recordings of research-grade and commercial products, which sometimes lack the capability of hardware synchronization via, e.g., transistor-transistor logic (TTL) (Iwama et al., 2022). LabStreamingLayer (LSL) is an often-used middleware ecosystem that enables the streaming and synchronizing of multiple data streams via the network (LabStreamingLayer, 2023). However, LSL can not take the hardware's intrinsic and the data transfer's delay and jitter into account. Thus, in cases where high temporal accuracy is required, those delays must be evaluated before the experiment (Artoni et al., 2018; Iwama et al., 2022). This study provides a method for measuring and understanding hardware offset and eventual limitations tailored toward an increasingly relevant combination of EEG, VR, and Eye tracking. We particularly emphasize general accessibility and flexibility through open-source solutions and the use of off-the-shelf VR-integrated eye tracking.

The objective of this study is, therefore, threefold:

1. Present a method for setting up a synchronized measurement of EEG and VR-headset-integrated eye movement and the subsequent real-time processing of the data streams.
2. Present a method for evaluating the temporal accuracy of the proposed setup.
3. Demonstrate a potential use case of the method using a hybrid SSVEP speller in a fully immersive VR environment.

## 2 Method

The following section provides an overview of the two setups used in this study. The first setup was utilized to calculate the latency and jitter of the VR-integrated eye-tracker, while the second setup was employed for the proof of concept SSVEP-Speller. For calculating the hardware latency and jitter of the VR-integrated eye-tracker, the temporal difference between the measured time points of complete eye closure detected by the eye-tracker and the EMG channel of the EEG amplifier was calculated. The calculated hardware offset is then used for the implementation of a proof of concept SSVEP speller, which uses the eye-tracker to make a pre-selection of a relevant subsection of a virtual 3D keyboard and the SSVEP response to select a specific letter.

### 2.1 Hardware latency evaluation

#### 2.1.1 Experimental setup

The experimental setup for the hardware latency analysis based on eye blinks comprised two computing devices, both running Windows: one desktop computer (PC1; AMD Ryzen 9 5950X, an RTX 3090, and 32GB of DDR4 RAM) running Unity and Neuropype, and one laptop (PC2) running EEG Recorder (Brain Vision Recorder) software.

##### 2.1.1.1 Set up for collecting electrophysiological data

**Figure 1:**
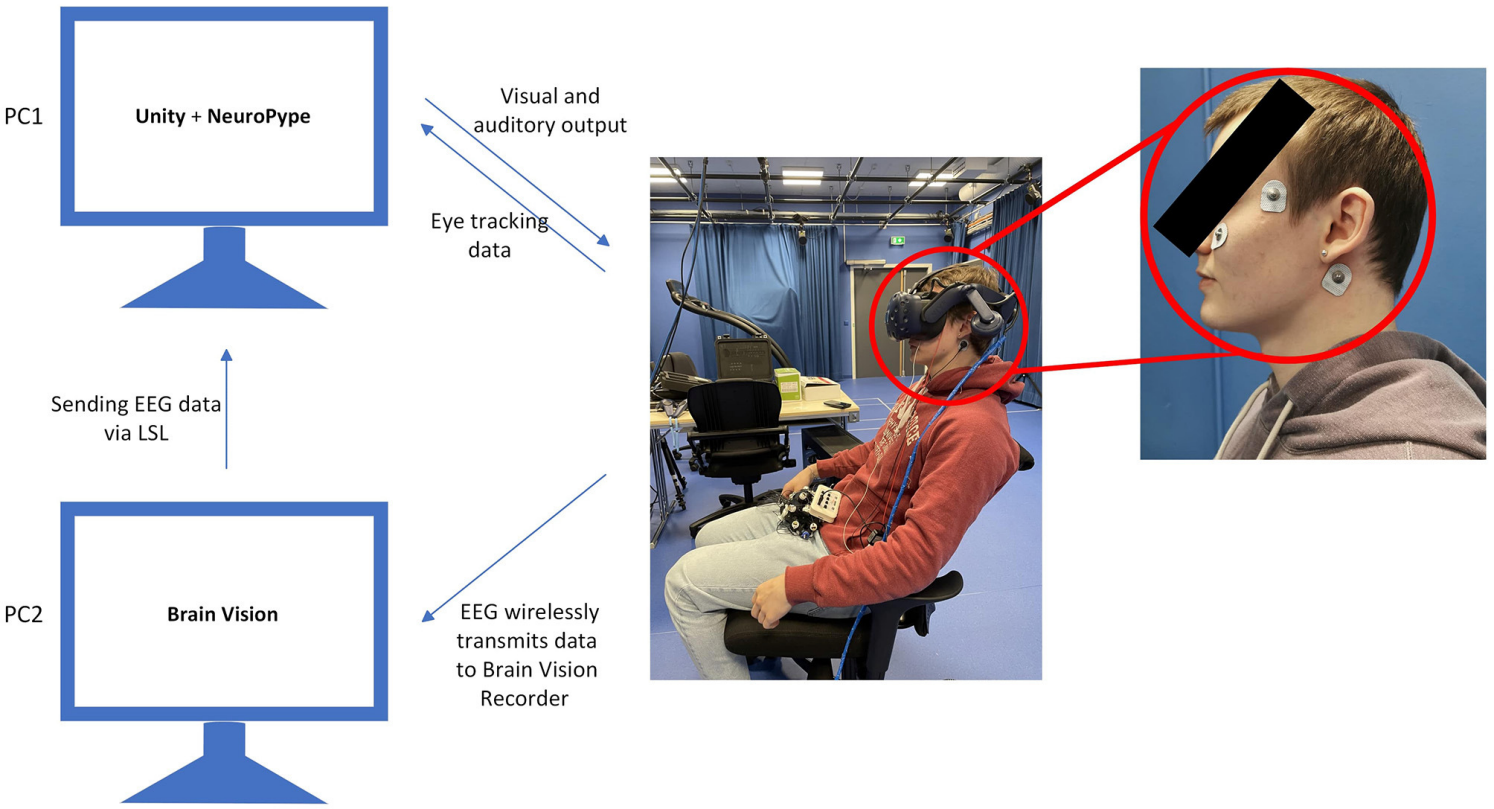
Experimental setup of the synchronization pipeline, showing EEG data being recorded by BrianProducts to PC2 and transferred from PC2 to PC1 using LSL. Simultaneously, eye-tracking data is collected from the VR headset and transmitted directly to PC1 via Unity. PC1 is responsible for visual and auditory output to the VR headset. The positions of the EMG electrodes are also shown, with the ground electrode placed under the right ear, while the recording electrodes are positioned beneath the left eye, one on the temple. The electrode placement was taken from a study by López (2015).

Electrophysiological data was collected using a Brain Products LiveAmp and Brain Products Trigger Extention Box with a single-channel EMG. The EEG channels were irrelevant for evaluating the hardware latency, as they were recorded using the same hardware. The EEG system operated at a sampling rate of 500 Hz. The participant wore three EMG electrodes at the following positions: one positioned beneath the left eye, one on the temple, and a ground electrode placed under the right ear (Figure 1). The EEG/EMG data stream was consistently transmitted using BrainProduct's LSLBrainAmpSeries (https://github.com/brain-products/LSL-BrainAmpSeries) at a sampling rate of 500 Hz from the EEG recorder. Wearing only the EMG and VR headset during synchronization was deemed necessary as the EEG data would not be utilized at this stage, resulting in the final setup seen in Figure 1.

##### 2.1.1.2 Setup for collecting behavioral data

Behavioral data was measured using a commercially available HTC VIVE Pro Eye VR headset with an integrated Tobii eye-tracker recording at a sample rate of 120 Hz. The eye-tracker's data stream was transmitted at a sampling rate of 250 Hz from the Unity software environment utilizing LSL4Unity integration (https://github.com/labstreaminglayer/LSL4Unity/, cloned April 2023). The eye-tracking data stream contained five channels of interest, with three of them representing binary values denoting the state of each eye (left, right, or both) in terms of closure, where a value of 1 signified complete closure. The remaining two channels provided decimal values indicating the degree of eye openness of each eye, with a value of 1 indicating complete eye aperture. This data was retrieved through the TobiiXR (v3.0.1) and SRanipal (1.3.6.8) APIs, respectively.

##### 2.1.1.3 Data handling

The LSL streams, transmitted from Unity (PC1) and the EEG recorder (PC2), were received and processed by PC1. Following this, a dejittering process was applied to these streams to remove irregularities in the timestamps of the data points (Intheon, 2022), facilitated by the dejittering function provided by NeuroPype (Academic Edition v2022.0.1). Subsequently, the processed data streams were saved into files formatted as Extensible Data Format (XDF), also using the NeuroPype software. As part of the data preprocessing, the EEG stream was downsampled from its original sampling rate of 500 Hz to 250 Hz before storing it. This downsampling operation was performed using the downsampling function within the NeuroPype software.

#### 2.1.2 Data acquisition

The dataset utilized in this study consists of 661 blinks and was obtained from 4 different participants, who received specific instructions to engage in natural blinking with both eyes. The participants were instructed to synchronize the eye blinks with a metronome at a tempo of 60 beats per minute (BPM). This was introduced to enhance the interpretability and ease of analysis of the recorded data.

Blink data was collected in sets of 10–20 consecutive blinks. Multiple sets of blinks were captured within a single recording session, and the eye-tracking system underwent recalibration between each successive recording session to maintain data accuracy and consistency. Each recording session captured around ten sets. A few blinks were excluded from the analysis due to instances where the EMG curve was distorted, or the blinks were considered outliers, with a value of more than three times the STD. An example of a distorted EMG curve (Figure S1) can be seen in the Supplementary data.

#### 2.1.3 Data analysis

Preprocessing of the EMG stream included filtering the EMG signal by applying a zero-phase Butterworth filter (0.75–5 Hz, 3^*rd*^ order) (Sharma et al., 2020). The entire analysis was performed using Python (3.11). The signal stream filtering achieves a signal curve that excludes frequencies outside the predefined frequency range. These frequencies would introduce noise and interfere with the accuracy of the signal analysis (Leske and Dalal, 2019). The zero-phase version was applied to correct any signal distortion created by the normal Butterworth filter (Leske and Dalal, 2019).

The synchronization algorithm operates by aligning two distinct data streams utilizing a standard biological marker, specifically the occurrence of an eye blink. This synchronization event coincides with the peak observed in the EMG signal and the initiation of a numeric value of “1” within the eye-tracker data stream, signifying the full closure of the eyes. The peak of the EMG curve was defined as the 90% amplitude maximum of the curve, allowing more reliable peak detection corresponding to a full closure of the eye. The TobiiXR API defines an eye as closed when the numeric value of openness is less than 0.1. It is crucial to emphasize that the analysis was conducted by comparing two data streams, both sampled at a rate of 250 Hz. However, it should be noted that due to the limitations inherent in the eye-tracker equipment, achieving a higher temporal accuracy than an index granularity of 8.33 ms (equivalent to 1/120th of a second) was unattainable. The blink recording files often contained varying quantities of samples. NeuroPype handled the recording of files, and different trimming strategies were employed to address this, where the strategy yielding the minimal STD was selected as the approach for file recordings of different lengths.

##### 2.1.3.1 Algorithm for finding the offset of a single blink

This algorithm was applied to every blink within each recording. The average offset from each recording session was compared to determine the final estimated offset value.

1. Input: Data of a single blink in two streams—EEG/EMG and eye-tracker.
2. Find the start index of the blink in the eye-tracker stream by locating the first numeric value of 1 in the both_blinking channel.
3. Filter the EMG channel in the EEG stream using the previously specified Butterworth filter.
4. Locate the index corresponding to the peak of the filtered EMG signal. To account for different slope lengths of the different blinks, the peak was set to 90% of the peak of the curve.
5. Calculate the difference between the indexes obtained in steps 2 and 4.
6. Multiply the index difference by the sampling rate to compute the offset in milliseconds.

##### 2.1.3.2 Algorithm to find mean offset of a series of multiple blinks

This algorithm was applied to all the valid blinks in every recording to find an average offset and variance between the EEG and eye-tracker.

1. Input: Eye tracking and EEG/EMG data as Python DataFrames.
2. Store index of every blink start by finding each sequence of 1's in the eye-tracking stream.
3. Split the DataFrames into pairs of both streams based on each individual blink and add some data points as padding.
4. Use the algorithm for finding the offset in a single blink, for every blink.
5. Use the different offsets to compute mean, STD, and average offset for the entire blink recording.
6. Do this for multiple recordings that contain multiple blinks and compute the offset.

### 2.2 Proof of concept—BCI speller

Based on the offset found for the synchronization, an SSVEP-based hybrid BCI speller was created in an immersive VR environment in Unity as a proof of concept. This demonstrates a potential use case for the synchronized equipment while being simple to implement. BCI spellers are widely employed BCI applications, encompassing diverse setups. This particular speller leverages SSVEP in conjunction with CCA, avoiding the need for the synchronization of the eye-tracker and EEG to have a millisecond-level precision (Bin et al., 2009; Zerafa et al., 2018). Furthermore, BCI spellers offer the advantage of available comparative results, making it easy to evaluate their performance. Notably, these applications are also relatively straightforward to implement, necessitating no more than a keyboard layout with flickering letters, in contrast to the requirements of more intricate gaming or application systems.

**Figure 2:**
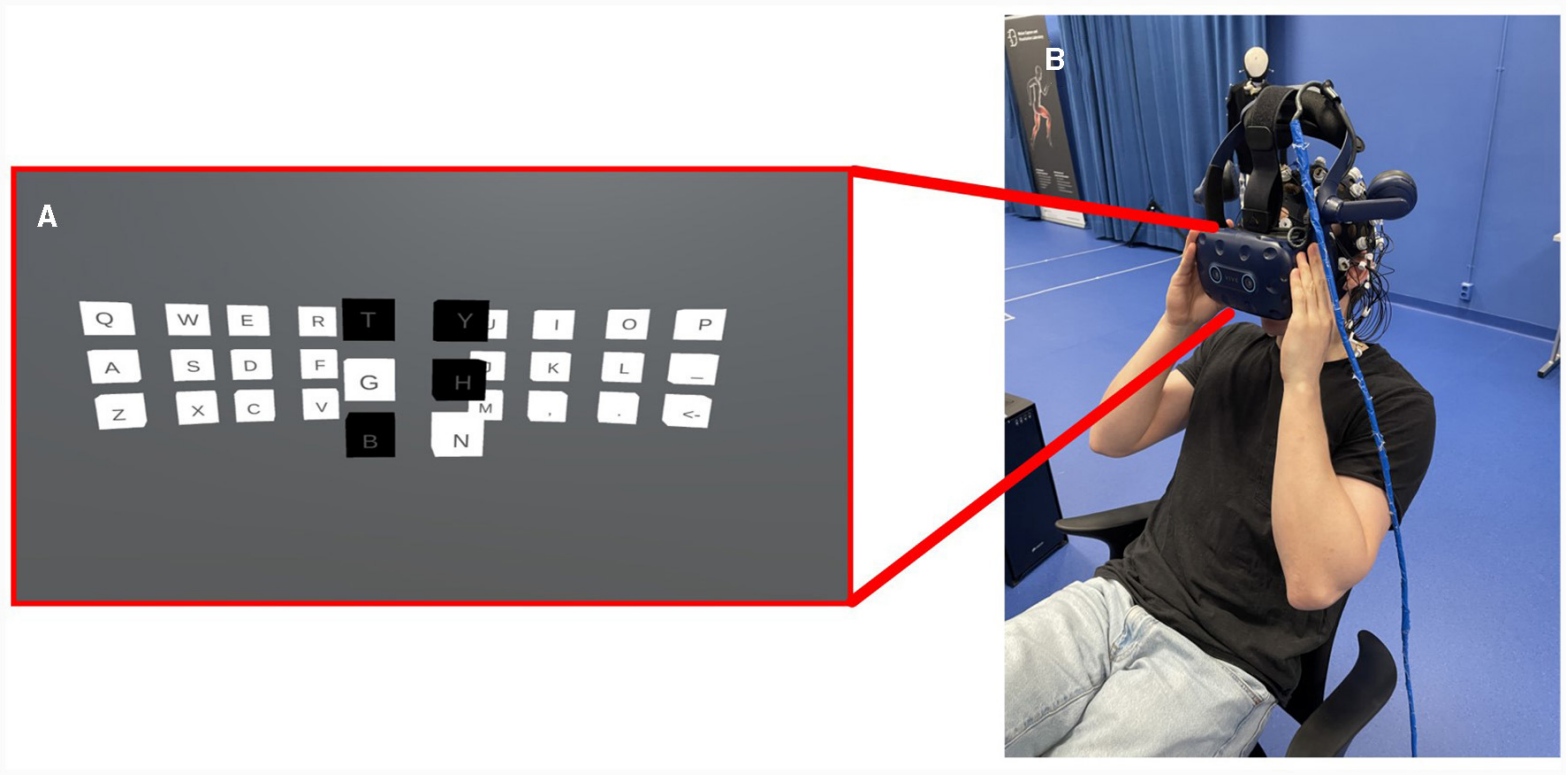
**(A)** Shows the speller setup in the immersive 3D VR environment where one of the clusters has been selected and has started to flicker. **(B)** Shows a person using the speller by holding it up to their face, making sure not to affect the most critical electrodes over the occipital region.

To accommodate the simultaneous use of the VR headset and the EEG recording equipment, the Fp1, F3, F7, F4, F8, and Fp2 electrodes were removed. Additionally, the ground electrode was repositioned to a higher location on the electrode cap. These adjustments were made to increase the comfort of the participant while wearing the VR headset and the EEG cap simultaneously. A participant might use the speller as shown in Figure 2B. The setup of the speller interface was inspired by Du et al. (2019) and Mannan et al. (2020), with clusters of letters that flew out toward the user of the speller when looked at. Mannan et al. (2020) states that the addition of an eye tracker makes the speller less tiring, and improves performance as compared to EEG only. The eye tracker and clusters enable the reuse of the flickering frequencies, only needing six frequencies for a six-letter cluster, contrary to 30 frequencies for 30 letters and symbols in the speller. Some differences were introduced, though. By taking a heuristical approach, the frequencies 4 Hz, 5 Hz, 5.5 Hz, 6 Hz, 7 Hz, and 7.4 Hz were chosen for the flickering of the letters, (1) due to lower frequencies eliciting stronger responses (Mannan et al., 2020) and (2) to avoid conflicting harmonization. Though the Information Transfer Rate (ITR) gets lower with lower frequencies, it was deemed ok considering the performance of the speller was not the main focus of this project. ITR is a common way to evaluate BCIs and says something about how effective the system is (Liu et al., 2012). Further, the layout of the keyboard was chosen to have a traditional QWERTY layout with some minor differences to the special characters (Figure 2A).

Some filtering techniques were implemented in a Python script and applied to remove noise and artifacts from the EEG. These include a notch filter (50 Hz) to remove the line nose, a band-pass filter (1–15 Hz) to give a reasonable threshold for the chosen flickering frequencies, and a zero-phase Butterworth filter of 3rd order with some initial padding that was eventually removed to return the signal to its original length. It should also be mentioned that the only electrodes used for the EEG signal were O1, Oz, O2, P3, P7, Pz, P8, and P4, as these are placed over the occipital region of the brain. These electrodes recorded data of the visual stimuli, which was later used to figure out which letter the participant was looking at.

The filtered EEG data, along with the reference signal that was calculated based on a data stream from Unity signaling when a cluster was flickering, was put in a Canonical Correlation Analysis (CCA) to calculate the similarities between the signals registered in the brain and the plausible flickering values. The calculation was based on the publication by Mannan et al. (2020) and is a robust way to calculate the correlation between some input signal and a given reference signal. This can be done without training data and without phase sensitivity (Zerafa et al., 2018).

It's essential to consider an ocular delay in the speller pipeline, which requires adjusting the EEG data accordingly. This delay will be added on top of the computed offset from the synchronization of the pipelines. This additional delay was set to 100 ms, which was retrieved from a study with a similar setup (Mannan et al., 2020), and is necessary due to the time it takes a visual signal entering the eye to be registered in the visual cortex (Li et al., 2015). When applying CCA to assess signal correlations, it's important to align the signals based on their arrival at the visual cortex rather than at the point when Unity displays the flickering. The speller was tested in two rounds with the same participants, though with a slightly modified setup in the latter round. Noteworthy improvement was evident during the initial testing phase, leading to the decision to keep the original speller configuration as the proof of concept.

## 3 Results

**Figure 3:**
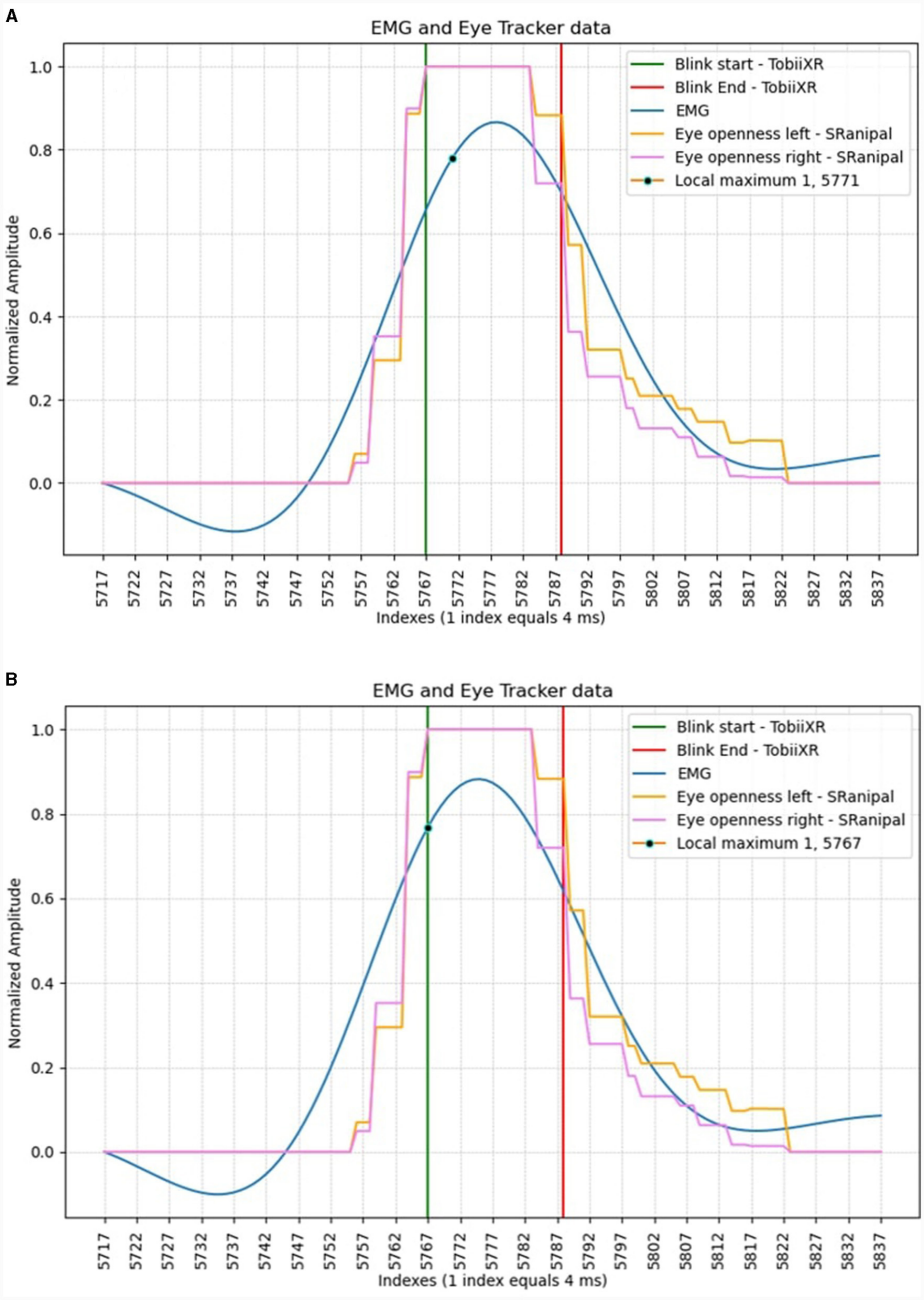
An eye blink recorded in both data streams before **(A)** and after **(B)**, adjusting for offset.

The following section presents the results from the analysis of the blinking data, showing the difference in the time it takes to sample a data point in the data streams, including the variance in the mentioned time. Then, the accuracy and Information Transfer Rate (ITR) found during testing of the BCI speller is presented. A graphical explanation of the data stream shifting can be seen in Figures 3A, B as before and after the shift, respectively.

The offset was computed on a dataset with 4 participants, with a cumulative total of 661 individual blinks considered for the analysis. A table containing the results for each participant (Table S2) and the results plotted in a histogram (Histogram S3) can be found in the Supplementary data.

### 3.1 Eye-tracker–latency and jitter

The data analysis method determined that the average offset between the EEG and eye-tracker is 8–10 indices, equating to 36 milliseconds with the given sampling rate. To be more precise, the eye-tracker was found to be 36 milliseconds faster than the EEG, and thus should be adjusted for this delay accordingly. The STD of the data at its lowest, measured 5.76 ms. This is lower than greater than a single sample (8.33 ms) when considering the limited sampling rate of the eye-tracker, which operates at 120 Hz.

### 3.2 BCI speller–accuracy and ITR

The speller was tested on 7 participants. Even with varying results in accuracy and Information Transfer Rate (ITR), some of the participants were able to use the speller with high accuracy for every flickering length, which supports the proof of concept that it is possible to utilize this combination in an immersive VR environment. The results of the speller's performance are presented in Table 1. The speller results showed higher accuracy for the longer flickering durations.

**Table 1:** The average ITR and accuracies for the test round for all tested flickering periods.

**Flicker period**	**Accuracy**	**ITR (bpm)**
6s	79.83%	25.26
5s	72.27%	24.79
4s	57.98%	20.43
3s	51.26%	20.71
2s	47.06%	23.85

## 4 Discussion

The primary strength of this study is the utilization and synchronization of two distinct and powerful off-the-shelf equipment setups with different data processing pipelines, which are all open source. The study demonstrates possible applications for the synchronized equipment by implementing a BCI application in an immersive VR environment, unlike some other variants such as EYE-EEG toolbox which is a bundle for synchronizing some standalone eye trackers with EEGs (EYE-EEG, 2021). Such combined usage has the potential to improve performances in a range of applications. For example, in software engineering, this technology can assess a programmer's comprehension, as demonstrated in eye-tracking studies (Lin et al., 2016). In engineering science, it measures cognitive load across tasks and participant knowledge levels (Keskin et al., 2020), identifies optimal learning approaches and efficiency (Baceviciute et al., 2022). Beyond engineering, applications include neuromarketing (Mashrur et al., 2023; Zhu and Lv, 2023), and safety training utilizing VR, eye tracking, and EEG to evaluate attitudes and learning abilities (Katona, 2014; Comu et al., 2021; Huang et al., 2022).

For the comparison of different synchronization methods, to our knowledge no study has yet presented a method for the synchronization of EEG and VR-integrated eye trackers, thus rendering a direct comparison of methods not applicable. There are nonetheless related studies that evaluate the general hardware delay of VR-integrated eye trackers. In a related study, researchers used the Vive Pro Eye with TobiiSDK and electrooculography (EOG) to quantify eye saccades, demonstrating a notable delay in the eye-tracking system (Stein et al., 2021). Their study revealed a mean offset of 50 ms latency in the eye-tracker, alongside an end-to-end delay of 80 ms within their experimental setup (Stein et al., 2021). Similar outcomes were identified in the present study, where the presented setup displayed an end-to-end offset of 36 ms. The apparent 44 ms variability could potentially be caused by transmission delays inherent in the differences in the experimental setup. Another aspect of the proposed experimental setup worth mentioning is the accessibility of the pipeline. This particular setup utilized an EMG for recording blinks in the EEG pipeline, but if no EMG is at hand, one could, for example, instead use the frontal electrodes of the EEG to record the same motions. This makes the experimental setup more accessible to a wider audience who might not have all the dedicated equipment to warrant synchronized usage. Given the increasingly widespread usage of BCI-VR systems with eye-tracking (Wen et al., 2021), there will be more cases where such synchronized usage is called upon. Our proposed method presents a flexible and affordable way of synchronizing the needed equipment.

The demand for precise synchronization varies from case to case. A study on the synchronization of EEG-EMG movements combined with motion capture found that even a 10 ms temporal misalignment between the devices can affect causal relationship investigations between EEG-EMG connectivity (Artoni et al., 2018). However, for less strict analyses, such as time-frequency transformations within 0.2 to 0.5-second time windows, synchronization demands can be more relaxed (Artoni et al., 2018). In Iwama et al. (2022), the authors attempted to integrate an EEG and an eye-tracker within an experimental setup similar to our study, and they underscored the importance of synchronized equipment in online applications such as the proof of concept speller, compared to offline analysis where one can adjust the signals accordingly. Large latency between different data streams fails to capture state-dependent differences in the streams, while significant jitter in the variable latency can be fatal if the application relies on external triggers, such as a P300 speller (Iwama et al., 2022; Kundu and Ari, 2022). In our study, after measuring the jitter of the setup, we chose a BCI speller based on SSVEP, which is less susceptible to the effects of jitter. It can be argued that an attempt to measure the latency and jitter between equipment should be advocated almost irrespectively because it can unearth and examine the underlining assumptions of hardware performances that subsequently confine and frame the architecture choices of the downstream applications.

Despite limitations in the sampling rate of the equipment, the proof of concept of the BCI Speller demonstrates the potential of the integration of an eye-tracker and an EEG. The speller drew inspiration from conventional SSVEP-based spellers with a keyboard layout. The speller also introduced an eye-tracker improvement by having fewer different flickering frequencies and placing the letters into clusters, resulting in reducing ocular and cognitive strain (Kundu and Ari, 2022). While a previous study has incorporated the combination of eye-tracking and EEG techniques in a BCI speller (Mannan et al., 2020), it is noteworthy that this has predominantly been confined to 2D computer screens. Furthermore, the development of BCI spellers within a VR environment has been documented, although without the inclusion of eye-tracking (Du et al., 2019). This study distinguishes itself because it combines a commercial EEG and a commercial VR headset with eye-tracking capabilities within a hybrid BCI Speller, set in a fully immersive VR environment. Both the accuracy and ITR of the presented BCI speller are lower to comparable state-of-the-art BCI spellers (Wen et al., 2021; Maslova et al., 2023). However, the primary intention of the study is to display the feasibility of the synchronized ecosystem, and this was also the main concern when implementing the speller. Our results indicate that longer flicker periods result in higher classification accuracy. This is to be expected as a higher sample count increases the certainty of the CCA's prediction (da Cruz et al., 2015). However, as longer flicker periods negatively impact the ITR, the length of the flicker period must be carefully balanced. Besides a speller, there are other applications that will benefit from the synchronized acquisition of EEG, and eye-tracking in a VR environment, such as observing human eye movement and brain activity during learning, social interactions, or other behavior studies that require an ecologically valid environment but are hard to study “in the wild”.

### 4.1 Limitations

The presented SSVEP-Speller is merely a proof of concept to show case possible application of synchronized EEG and VR-integrated eye tracking. It was chosen due to its robustness and simplicity compared to e.g., P300-Spellers. Retrospectively, our SSVEP-Speller is not highly dependent on perfect hardware offset compensation between the eye tracker and EEG. We, thus, strongly encourage future research to investigate applications that are more sensitive to correct hardware offset correction, such as fixation-related potentials (Kamienkowski et al., 2012). Further, the impact of correct hardware offset correction, should be quantified before and after offset adjustment. The presented method for hardware offset and jitter measurements, however, is suitable for different applications and experimental setups, due to its flexibility.

It is also worth noting that this is a method paper, to show how ET and EEG can be synchronized in VR, the exact number of offsets we arrive at is of less importance, as it will likely differ when other people use different hardware and setups. Future applications should repeat the process, not just use the offset we arrive at, due to hardware and inter-subject differences.

## 5 Conclusion and future scope

This study successfully demonstrates the possibility of synchronizing the data streams of commercially available equipment, in an accessible and easy-to-use manner with synchronization results on par with similar setups, with a computed jitter of less than 9 ms. To demonstrate the usability of such synchronized equipment an online hybrid SSVEP-based BCI Speller was implemented as a proof of concept. It is important to note, however, that the method presented is developed for accessibility, and may not be well-suited for more sophisticated EEG and eye-tracking applications. Furthermore, the calculated STD for both data streams exceeds a single sample by 0.44 ms. To find the limitations of this method a more exhaustive investigation using equipment characterized by a higher sampling rate is encouraged.

## Data availability statement

Publicly available datasets were analyzed in this study. The repository for the Unity implementation of the BCI Speller and the Unity Blinking environment: https://gitlab.stud.idi.ntnu.no/group-92/eye-tracking-unity-lsl. The repository for the NeuroPype pipelines the analysis of the blinking data, and the CCA analysis for the speller: https://gitlab.stud.idi.ntnu.no/group-92/neuropype-pipeline. YouTube link that showcases the speller in use, spelling “HEI”: https://www.youtube.com/watch?v=s6PwwigH5AA.

## Ethics statement

The studies involving humans were approved by SIKT–Norwegian Agency for Shared Services in Education and Research (975161). The studies were conducted in accordance with the local legislation and institutional requirements. The participants provided their written informed consent to participate in this study. Written informed consent was obtained from the individual(s) for the publication of any potentially identifiable images or data included in this article.

## Author contributions

OL: Formal analysis, Investigation, Methodology, Software, Validation, Visualization, Writing—original draft, Writing—review & editing. WT: Formal analysis, Investigation, Methodology, Software, Validation, Visualization, Writing—original draft, Writing—review & editing. EL: Conceptualization, Methodology, Project administration, Resources, Supervision, Writing—review & editing. TH: Project administration, Resources, Writing—review & editing. GS: Project administration, Resources, Writing—review & editing. TIH: Writing—review & editing. XS: Project administration, Resources, Writing—review & editing. AH: Project administration, Resources, Writing—review & editing, Conceptualization, Methodology, Supervision.
