# Supplementary material for: A method for synchronized use of EEG and eye tracking in fully immersive VR
Source: Front Hum Neurosci. 2024 Feb 26;18:1347974. doi: 10.3389/fnhum.2024.1347974 (PMC10925625; doi:10.3389/fnhum.2024.1347974)
Supplement: Supplementary file 1 [file Data_Sheet_1.pdf]

Supplementary Data

Figure S1: Example of distorted blink removed from the analysis

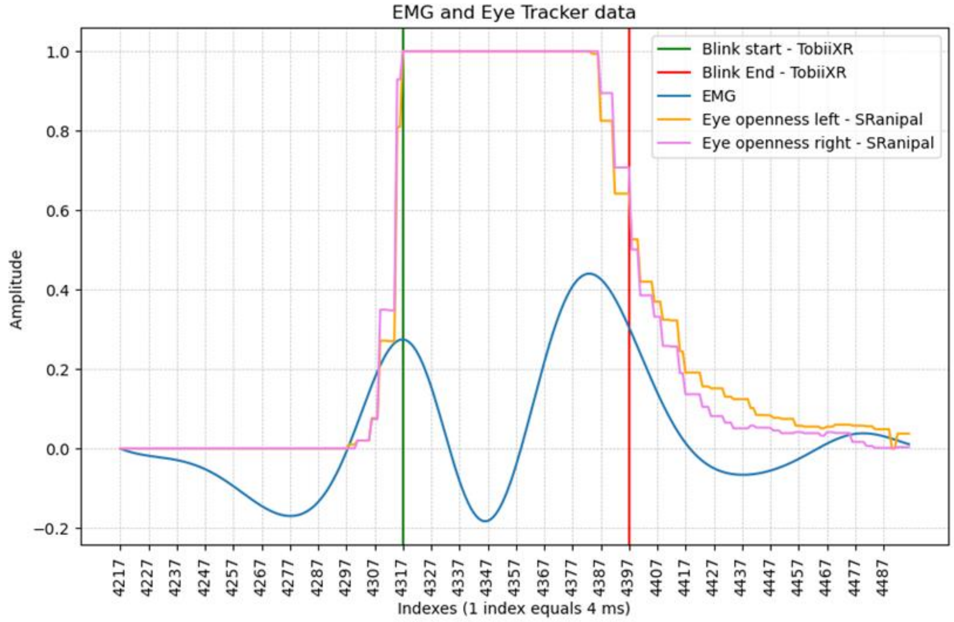

Example of a distorted blink in the EMG, which was removed from the analysis. The blink should have been a single slope as can be seen in Figure 3 in the paper. This is not a typical blinking wave usually seen in EMGs, and therefore discarded.

Table S2: Table of additional data collected

Participant	Offset (ms)	Jitter (ms)	n blinks
1	-36	5.89	171
2	-26	4.43	215
3	-47	5.71	275
4	-34	7.02	119
**Average**	**-36**	**5.76**	**195**
**Sum**			**661**

An additional round of data collection (with 4 subjects each about 200 blinks) for the calculation of the hardware offset (mean) and jitter (standard deviation) was conducted in January 2024.

Histogram S3: Histogram of computed offset based on data collected

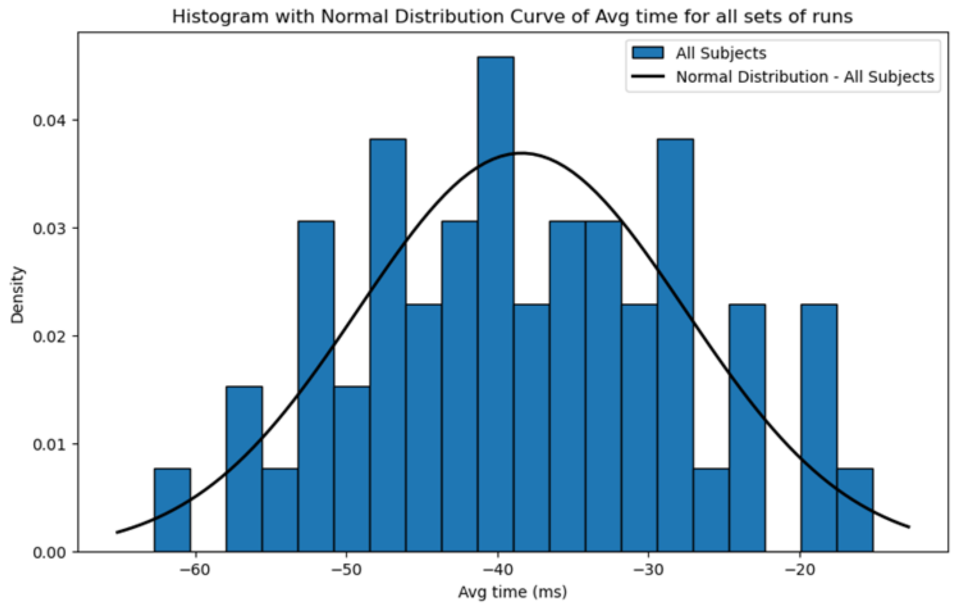

Histogram containing the different offsets found in the different recordings from the 661 blinks by the 4 participants. Used to find the offset between the EEG and the ET.
